# Cultivation of gut microorganisms of the marine ascidian *Halocynthia roretzi* reveals their potential roles in the environmental adaptation of their host

**DOI:** 10.1007/s42995-022-00131-4

**Published:** 2022-04-26

**Authors:** Yang Yang, Yuting Zhu, Haiming Liu, Jiankai Wei, Haiyan Yu, Bo Dong

**Affiliations:** 1 Ministry of Education Key Laboratory of Marine Genetics and Breeding, College of Marine Life Sciences, Ocean University of China, Qingdao, 266003 China; 2 Laboratory for Marine Biology and Biotechnology, Pilot National Laboratory for Marine Science and Technology (Qingdao), Qingdao, 266237 China; 3 Institute of Evolution and Marine Biodiversity, Ocean University of China, Qingdao, 266003 China

**Keywords:** Aerobic culture, Anaerobic culture, Ascidian, Defense, Gut microorganism

## Abstract

It has long been known that abundant symbiotic bacteria exist in the tunic and gut of marine ascidians, and that these play crucial roles in host development, physiological metabolism, and environmental adaptation. However, the identity, roles and functions of these symbiotic bacteria are known for only a few strains. In this study, we isolated and cultivated 263 strains of microorganisms from the intestine of the marine ascidian *Halocynthia roretzi* through a combination of aerobic and anaerobic culture approaches. Most cultivated species, both aerobic and anaerobic, from ascidian stool samples belonged to the genus *Bacillus* based on 16S rDNA sequencing identification and phylogenetic assays. The distribution of cultured bacteria varied with seasonal changes in environmental conditions. To explore the functions of cultured bacteria, we screened out a strain of *Serratia* sp. whose extracts showed high antibacterial activity against aquatic pathogens. These findings revealed the potential roles of gut microorganisms in ascidian defense and environmental adaptation, thus providing insights into the interaction and co-evolution between gut bacteria and their hosts.

**Supplementary Information:**

The online version contains supplementary material available at 10.1007/s42995-022-00131-4.

## Introduction

The intestinal microbiome of animals is closely related to the physiological processes, nutrition, and metabolism of the host (Human Microbiome Project 2012; Sommer and Bäckhed 2013). Recent studies have demonstrated that this microbiome could confer benefits on the host by improving resistance to colonization by pathogens, influencing the structure and function of the intestine, promoting the development of the immune system, and increasing the efficiency of energy absorption from food. For example, microbes in the gut of *Hydra vulgaris* provide short-chain fatty acids, thus helping the host to inhibit the reproduction of pathogenic microorganisms (Fraune et al. 2015). The host in turn provides adequate nutrition and suitable living conditions for, and influences the structure and composition of, the microbial community (David et al. 2014).

Marine ascidians belong to the subphylum Urochordata, which occupies a pivotal position in chordate evolution. Several ascidian species have become model organisms for studies of developmental and evolutionary biology. The microbiome of ascidians has drawn considerable attention in recent years. A large number of natural compounds derived from ascidian symbionts show high bioactivity and some exhibit anti-cancer activity (Dou and Dong 2019), suggesting their therapeutic potential in cancer treatment (Crawford and Clardy 2011).

Similar to vertebrates, the intestines of ascidians contain numerous microorganisms (Dishaw et al. 2012). Further studies showed that the ascidian gut is covered by a membrane that structurally resembles invertebrate peritrophic membranes in that both are framed with chitin nanofiles which gut microorganisms colonize (Nakashima et al. 2018). There is a unique bacteria-host interaction pattern in the intestine of ascidians. Studies have shown that geographically isolated ascidian populations have the same core flora (Dishaw et al. 2014), indicating that ascidians have a unique gut microbiota. These microorganisms have various unexpected functions in maintaining and regulating the physiology of host animals and improving environmental adaptation (Sommer and Bäckhed 2013). For example, as filter-feeding organism, ascidians are in contact with a large number of external pathogenic microorganisms, and yet have to acquire beneficial bacteria, which is a huge challenge. Studies have pointed out that ascidians can actively secrete variable region-containing chitin-binding proteins that help beneficial bacteria to settle in their intestine. These bacteria participate in the gut immune activity of the host (Dishaw et al. 2016). The diversity-generating metabolisms from both microbiota and host might lead to co-evolution and environmental adaptation (Wei et al. 2020a, b).

Ascidian gut microorganisms have been investigated by metagenomic and amplicon methods (Schmidt 2015). To further explore and validate the roles and functions of gut microbiota, the culture of intestinal bacteria is a technique that can still be employed although only a small proportion (0.01–1%) has been successfully cultivated. Our previous work on aquaculture animals characterized the gut microbial communities of the ascidian *Halocynthia roretzi* across the changes of season. The results revealed that *H. roretzi* harbors an indigenous gut microbiome that is distinctly different to the marine environmental microbial community and that there are significant seasonal variations in the composition and abundance of gut bacteria, with predominant bacterial orders representing each season (Wei et al. 2020a, b). In addition, we observed that the abundance of phytoplankton in the waters of the aquaculture area where the *H. roretzi* specimens were collected was subject to significant seasonal variation (Wang et al. 2008). Therefore, in the present study, we collected specimens of *H. roretzi* from the same aquaculture area in each of the four seasons of a year and performed isolation, culture and functional assays on their gut bacteria. During the study, we obtained 263 strains of ascidian gut bacteria through a combination of anaerobic and aerobic culture. The microorganism with antibacterial ability was screened out. The bioactivity of its extract was validated, thus providing evidence to support the assertion that the gut microbiome plays an active role in environmental adaptation.

## Results

### Aerobic culture of ascidian gut microorganisms

**Fig. 1:**
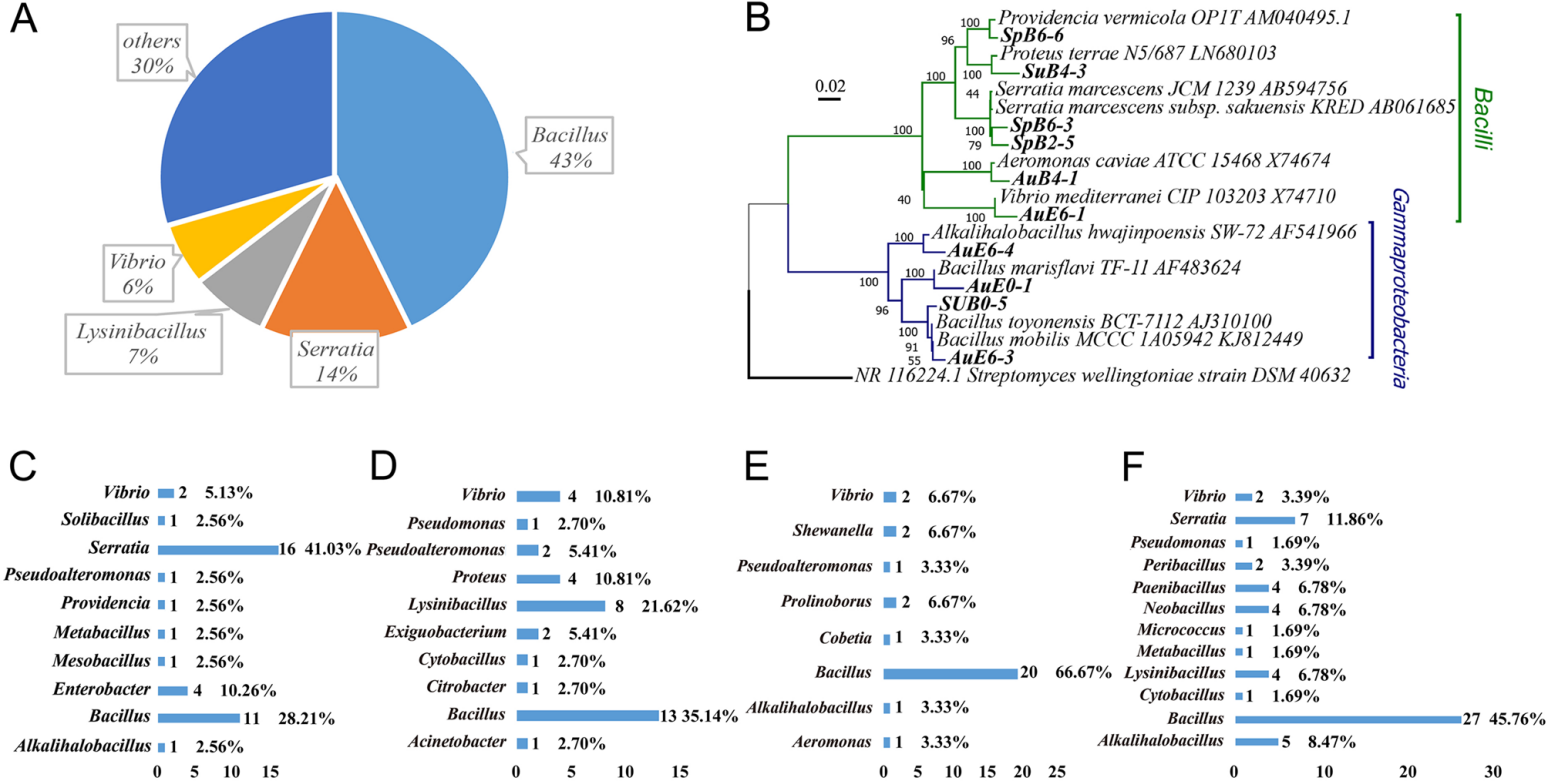
The diversity of culturable aerobic microorganisms in the gut of *Halocynthia roretzi*. **A** Diversity of culturable microorganisms obtained from aerobic culture of the samples. **B** Evolutionary tree of bacterial strains in aerobic culture. Novel strains are in bold font. *Streptomyces wellingtoniae* is the outgroup. **C**–**F** Number and proportion of culturable strains obtained from aerobic culture in spring (**C**), summer (**D**), autumn (**E**), and winter (**F**) samples, respectively

After aerobic culture and sequencing identification, a total of 165 strains of bacteria were obtained from ascidian gut samples. The cultured aerobic bacteria could be divided into six classes (Gammaproteobacteria, Actinobacteria, Bacilli, Deltaproteobacteria, Clostridia, and Flavobacteriia), 12 orders, 23 families, and 25 genera. The diversity of the culturable microbial community was high in each season with a Shannon index > 2.5 in each season (Table S1). The most culturable microorganisms obtained (a total of 71 strains) belonged to the genus *Bacillus*, accounting for 43% of all cultivated aerobic microorganisms (Fig. 1A). The second most culturable microorganisms obtained (a total of 23 strains) were members of the genus *Serratia*, accounting for 14% of all cultivated aerobic microorganisms. The third most culturable microorganisms obtained (a total of 12 strains) were members of the genus *Lysinibacillus*, accounting for 7% of all cultivated aerobic microorganisms (Fig. 1A). Among the obtained strains, 10 had a genetic identity of < 97% compared with reference strains and were therefore considered as potential new species. The phylogenetic tree showed that all these potentially novel bacterial strains belonged either to the class Gammaproteobacteria or the class Bacilli (Fig. 1B).

The ascidian gut samples were collected during each of the four seasons of a year. Among the culturable microorganisms, 39 strains classified into nine genera were obtained during spring (Fig. 1C), 37 strains classified into 10 genera were obtained during summer (Fig. 1D), 30 strains classified into eight genera were obtained during autumn (Fig. 1E), and 59 strains classified into 11 genera were obtained during winter (Fig. 1F).

Among the culturable ascidian gut bacteria, the genera *Bacillus* and *Vibrio* occurred in samples collected during each of the four seasons. The genus *Bacillus* was the most abundant representing 28%, 35%, 67%, and 46% of the total culturable microbiome in spring, summer, autumn and winter, respectively (Fig. 1C–F). Although members of the genus *Bacillus* were the most abundant bacteria overall, *Serratia* had the highest abundance during spring accounting for 41% of culturable microorganisms (Fig. 1C). These data indicated that the dominant populations of bacteria from the ascidian gut were relatively stable, whereas some strains were seasonally dependent. For example, *Cytobacillus* only appeared in summer and not in any other season whereas *Alkalihalobacillus* appeared in spring, autumn, and winter, but was absent in summer.

### Anaerobic culture of ascidian gut microorganisms

**Fig. 2:**
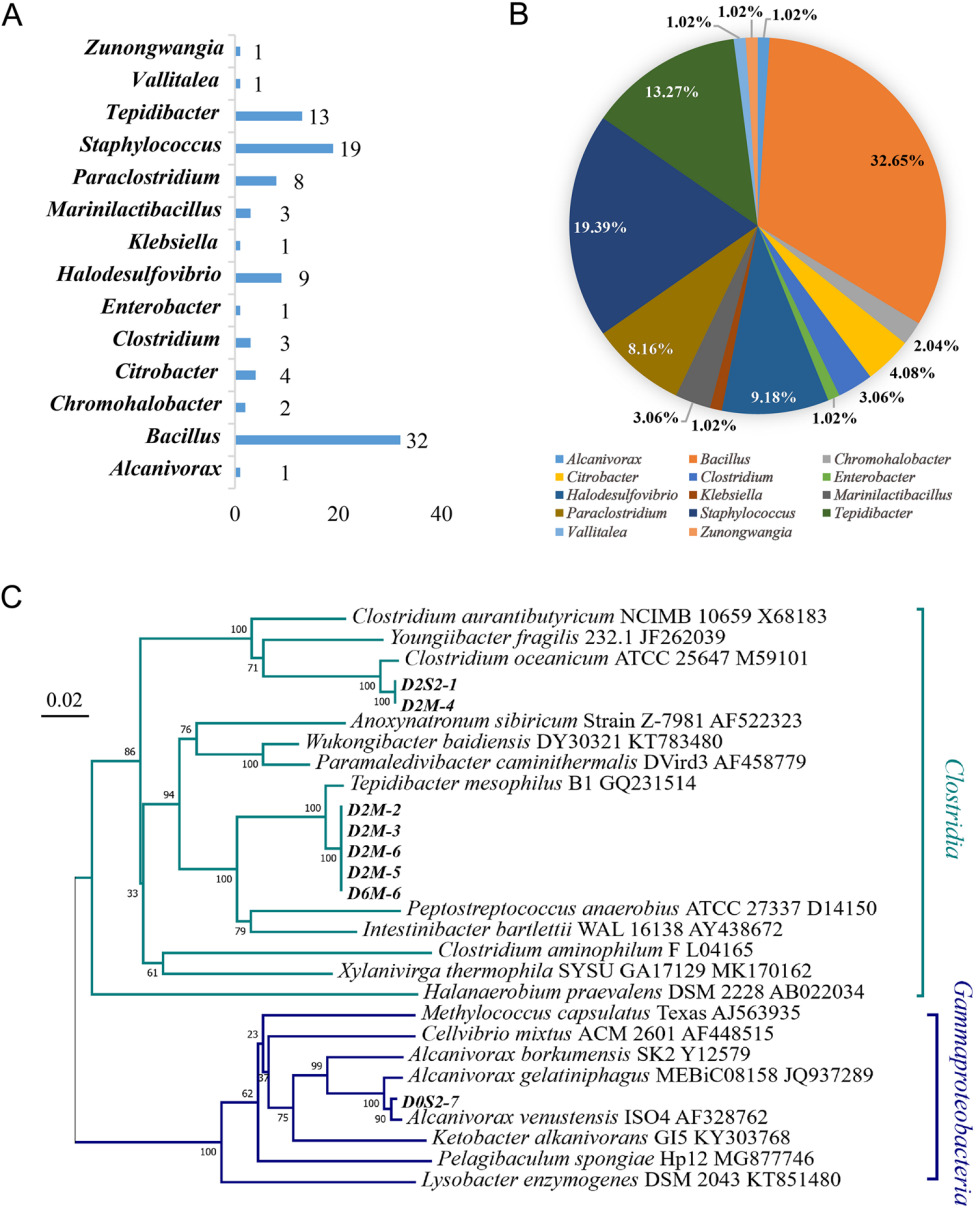
The diversity of culturable anaerobic microorganism in the gut anaerobes of *H. roretzi*. **A** Diversity of cultivable anaerobic microorganisms. **B** Proportions of cultivable anaerobic microorganisms. **C** Evolutionary tree of bacterial strains in anaerobic culture. Novel strains are in bold font.

We also performed anaerobic culture of ascidian gut bacteria. Because of the limitation of space and equipment, samples from only one season, i.e., winter, could be processed by anaerobic cultivation. A total of 98 strains of anaerobic culturable microorganisms belonging to 14 genera were recovered (Fig. 2A, B). The genus *Bacillus,* with 32 strains, was the most abundant, accounting for 32.65% of the total, followed by *Staphylococcus*, which accounted for 19.39%. The new strains were classified based on the 16S rDNA sequencing and all of these belonged to one of two classes, i.e., Gammaproteobacteria or Clostridia (Fig. 2C).

### Bioactivity detected from an ascidian gut bacterium

**Fig. 3:**
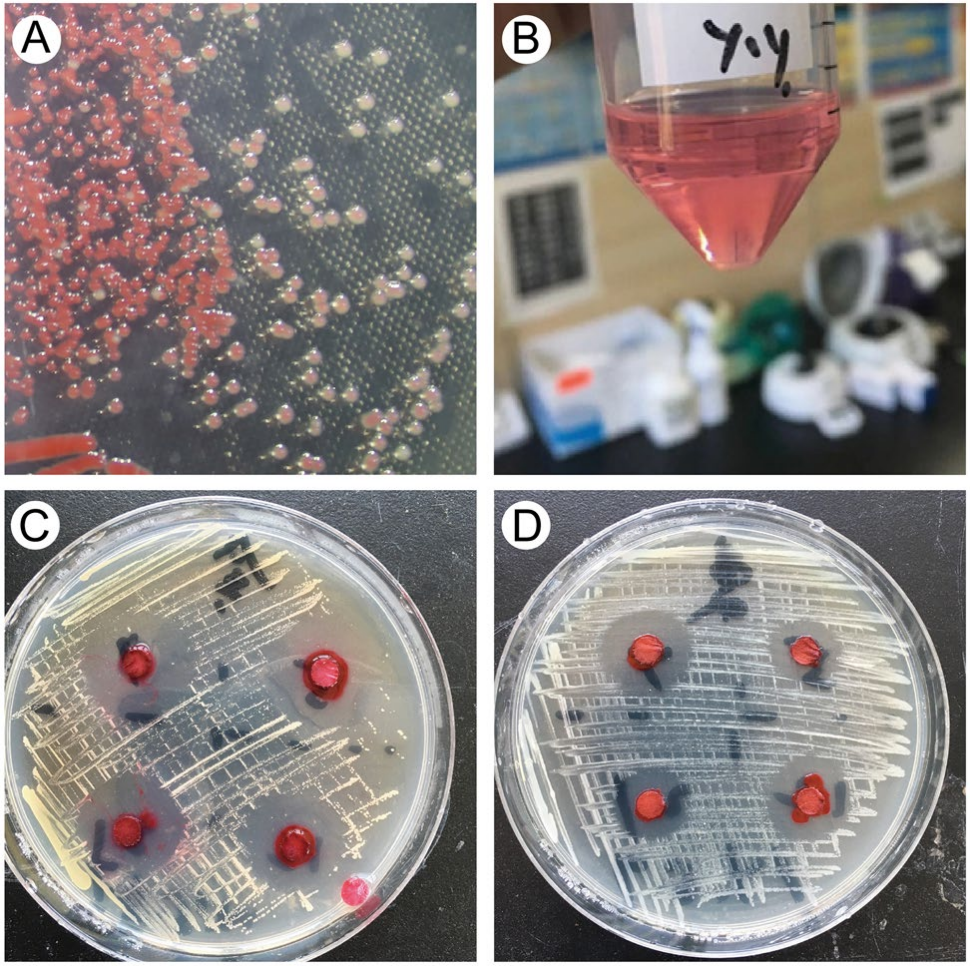
Biological activity of *Serratia* sp. **A** Colonies of *Serratia* sp. Red color appeared in colonies growing in high density. **B** Crude extracts from *Serratia* sp. **C** The growth of *Staphylococcus aureus* was inhibited by the extracts. **D** The growth of *Proteus* sp. was inhibited by the extracts

During cultivation of the gut bacteria, we observed inhibitory phenomena around one type of bacterium, and the gradual appearance of a red color in its colonies which grew in high density (Fig. 3A). These observations indicated the potential antibacterial activity of this bacterium. We therefore performed a genus-level identification and showed that it is a member of the genus *Serratia*. We further conducted a rough extract from colonies showing a red color (Fig. 3B) and selected aquatic pathogenic bacteria to examine the antibacterial effects of the extracts. The results showed that these extracts have high inhibitory effects on both gram-positive (*Staphylococcus aureus*) (Fig. 3C) and gram-negative (*Proteus* sp.) bacteria (Fig. 3D).

## Discussion

As fouling organisms, ascidians display adaptations to a wide range of environmental conditions. Recent studies have shown that the expansion of the ascidian genome potentially affects their environmental adaptability (Dou and Dong 2019; Wei et al. 2020a, b). Some studies also hint at the contribution of gut microbes to the environmental adaptability of ascidians, however most recent studies on intestinal microbes of ascidians were limited to species composition. Hitherto, only a few gut bacteria strains have been cultivated, and germplasm resources have not been preserved. In the present study, we investigated the gut microbes of *H. roretzi*. By using aerobic and anaerobic culture methods, a variety of microbial species were isolated and identified. Furthermore, we found that the observed community structure in ascidian gut varied with the culture methods used. For example, *Vibrio* was found exclusively in aerobic culture rather than anaerobic culture. However, some species could survive in different conditions. For example, the genus *Bacillus* could be cultured by both aerobic and anaerobic approaches no matter in which season the samples were collected. This suggests that genus *Bacillus* might be a core component of the gut microbiome of *H. roretzi* and potentially plays an important role in promoting adaptation of its ascidian host to diverse environmental conditions.

Members of the genera *Bacillus* and *Vibrio* have been previously isolated from the gut of ascidians (Utermann et al. 2021)*.* The class Gammaproteobacteria has a high abundance in the intestine of ascidians (Dishaw et al. 2014; Utermann et al. 2021). In *H. roretzi*, we noticed that different strains appeared during culture processes in different seasons. For example, the abundance of the genus *Serratia* in spring was significantly higher than that in other seasons. This may be due to changes in the sea water environment because the host adapts to different external environments by adjusting the structure of its intestinal microbial community (Wei et al. 2020a, b). For example, the breeding environment varies according to the temperature, salinity, nutrient levels, etc. in different seasons, which could affect the structure of the ascidian intestinal microbial community. The multiple biological activities of *Serratia* helps the host adapt to these changing environments (D'Alessio et al. 2000). The findings of the present study suggest that *Serratia* can help ascidians to resist harmful bacteria when the temperature increases in spring and seawater microorganisms begin to proliferate rapidly.

The present study also demonstrated that the ascidian gut microbiome is seasonal since the community structure of the cultured bacteria was distinct for each season. For example, the genus *Desulfovibrio,* which was previously reported to perform free nitrogen fixation under anaerobic conditions (Sayavedra et al. 2021), appeared in the cultures of samples collected in winter. It is noteworthy that there is limited nutrient availability during the winter (Fu et al. 2009). Therefore, the nitrogen-fixing activities of *Desulfovibrio* in the intestine may provide a beneficial effect on the adaptation of the host to winter conditions by mitigating the effects of nutrient deficiency.

*Halocynthia roretzi* has a close association with microorganisms. Numerous bacteria of the genus *Bacillus* were isolated in the present study, many of which serve as probiotics in common aquatic organisms (Yan et al. 2014). *Bacillus* is known to inhibit the growth of harmful bacteria, degrade organophosphorus and aflatoxin, and enhance host immunity (Jiménez et al. 2013; Liu et al. 2020). *Proteus* has been reported to exert nitrate ammonification effects, and *Acinetobacter* has denitrification effects, suggesting these genera may have a role in nitrogen circulation in the intestine of ascidians (Behrendt et al. 2015; Van Veen et al. 1993). These probiotic bacteria not only inhibit pathogenic microorganisms in the intestine (Walker and Baldridge 2019), but also stimulate and strengthen the immune system of the gut mucosa (Geuking et al. 2011; Round et al. 2011; Shaw et al. 2012). Immune regulation occurs in the surface of the gut mucosa in teleosts and plays a role in defending against pathogens (Tan et al. 2019). In this way, the immune response helps to regulate the microbial community in the gut (Ngamkala et al. 2010; Pessione 2012). On the other hand, the present study also found that in the intestine of *H. roretzi*, bacteria such as *Serratia* produce metabolites that are of importance in resisting the invasion of foreign microorganisms. Furthermore, bacteria of the genus *Desulfovibrio*, some species of which can bind free nitrogen under anaerobic conditions (Behrendt et al. 2015), were also isolated in the present study. It is therefore possible that in food- and nutrient-limiting conditions, these nitrogen-fixing activities can alleviate the nutrient-depleted environment in the intestines of *H. roretzi* and help maintain the survival of the animal. These results suggest that there is a beneficial symbiotic relationship between ascidians and their gut bacteria, which probably co-evolved over a long period of time.

*Halocynthia roretzi* not only has high economic value as an edible marine animal, but is also viewed as a source of natural products with medicinal properties. For example, *H. roretzi* accumulates vanadium compounds with effective anti-diabetic activity (Gunasinghe and Kim 2018), and its edible partial hydrolysate can induce apoptosis of human colon cancer HT-29 cells by activating reactive oxygen species (Oh et al. 2019). There is also evidence that a large number of gut symbiotic microorganisms could facilitate the ascidian to secrete antibiotics as a defense against external pathogens (Wei et al. 2020a, b). And in the present study, we have demonstrated that ethanol extracts of *Serratia* sp. isolated from the gut of *H. roretzi* has anti-bacterial properties. These findings provide evidence for the importance of ascidian gut microorganisms to the survival of their host.

## Materials and methods

### Animals and sample collection

Adult specimens of the ascidian *H. roretzi* were collected from an aquaculture farm in Weihai City (37.17713° N, 112.5742° E), Shandong Province, China, in January, April, July, and October 2018. The animals were rinsed with sterile water and then put in a sterile environment. Disinfecting apparatus (75% alcohol immersion) was employed to dissect and open the atrial siphon thereby allowing access to the intestinal tract. Stools were collected and stored into a sterile Eppendorf tube pre-filled with seed preservation solution (20% sterile glycerol). After freezing in liquid nitrogen, the samples were stored at − 80 °C until the culture experiments could be conducted. During the cultivation, all operations were carried out in an ultra-clean environment to avoid contamination. Only a single sample was used in each operation to avoid cross contamination.

### Aerobic culture

100 μl of the stored sample was added to 2 ml Marine Agar Zobell 2216 medium (MA; 5 g Bacto peptone, 1 g yeast extract and 0.01 g FePO_4_ in 1 L seawater) (HB0132, Hepobio, Qingdao) or Brain–Heart Infusion Broth (BHI) (HB8297-5, Hepobio, Qingdao) liquid medium and incubated at 20 °C for 24 h. The cultures were then diluted serially 100, 1000, or 10,000 times, and 200 μl of each diluted culture was spread on either MA, BHI, Yeast Malt Agar (YMA) (M2323, Tuopu Biotechnology, Shandong), or Ashby's mannitol agar (Ashby) (HB8540, Hepobio, Qingdao), solid medium plates for aerobic culture at 20 °C for 48 h or 72 h. Single colonies were picked for DNA extraction and 16S rDNA sequencing for species identification.

### Anaerobic culture

Using an anaerobic operation box, a 100 μl aliquot comprising 90 μl glycerin and 10 μl of the stored sample was added to 2 ml MA or South Pacific Gyre (SPG) (D’Hondt et al. 2011) liquid medium and incubated at 20 °C for 24 h. The culture was diluted 100, 1000, or 10,000 times, respectively. Then 200 μl of the diluted culture was spread onto MA or SPG plates and incubated at 20 °C for 48 or 72 h in anaerobic conditions. Single colonies were picked for DNA extraction and 16S rDNA sequencing for species identification.

### Species identification and construction of phylogenetic tree

Genomic DNA was extracted from a fresh culture of the selected colony based on a previously reported protocol (Gontang et al. 2007). Cloning of the 16S rRNA gene was carried out using the universal primers B8F (5′-AGAGTTTGATCCTGGCTCAG-3′) and 1510R (5′-GGTTACCTTGTTACGACTT-3′). Sequence alignment and species identification were performed on the EzTaxon server (Yoon et al. 2017). Phylogenetic trees were constructed using the neighbor-joining (NJ) algorithm (Saitou and Nei 1987), with bootstrap values calculated from 1000 repetitions of executions using the routines included in the Molecular Evolutionary Genetics Analysis (MEGA) software. The sequencing data used in the construction was from List of Prokaryotic names with Standing in Nomenclature (LPSN 07/08/2021) (Parte et al. 2020).

### Examination of antibacterial activity of *Serratia *sp*.*

The antibacterial activities of *Serratia* sp*.* were examined by zone-of-inhibition experiments. *Staphylococcus aureus* and *Proteus* sp. were used for the antibacterial experiments. *Staphylococcus aureus* and *Proteus* sp. were cultured on solid LB medium (HB0128, Haibo, Qingdao). Sterile filter paper with a diameter of 6 mm was carefully placed on the surface of the solid LB medium. LB bacterial culture medium was put in the middle of the filter paper, and the plates were then cultured at 26 °C for 24 h before examination.

## Supplementary Information

Below is the link to the electronic supplementary material.

Supplementary file1 (DOCX 29 KB)

## Data Availability

All data generated or analyzed during this study are included in the manuscript and supporting files.
