# Supplementary material for: Cultivation of gut microorganisms of the marine ascidian Halocynthia roretzi reveals their potential roles in the environmental adaptation of their host
Source: Mar Life Sci Technol. 2022 Apr 26;4(2):201–7. doi: 10.1007/s42995-022-00131-4 (PMC10077266; doi:10.1007/s42995-022-00131-4)
Supplement: Supplementary file 1 — Supplementary file1 (DOCX 29 KB) [file 42995_2022_131_MOESM1_ESM.docx]

**Supplementary data**

**Cultivation of gut microorganisms of the marine ascidian *Halocynthia roretzi* reveals their potential roles in the environmental adaptation of their host**

Yang Yang^1^, Yuting Zhu^1^, Haiming Liu^1^, Jiankai Wei^1^, Haiyan Yu^1,*^, Bo Dong^1,2,3,*^

^1^ Ministry of Education Key Laboratory of Marine Genetics and Breeding, College of Marine Life Sciences, Ocean University of China, Qingdao, 266003, China

^2^ Laboratory for Marine Biology and Biotechnology, Pilot National Laboratory for Marine Science and Technology (Qingdao), Qingdao, 266237, China

^3^ Institute of Evolution and Marine Biodiversity, Ocean University of China, Qingdao, 266003, China

* Corresponding Authors: Haiyan Yu ([haiyanyu@ouc.edu.cn](mailto:haiyanyu@ouc.edu.cn)); Bo Dong ([bodong@ouc.edu.cn](mailto:bodong@ouc.edu.cn))

**Table S1. The classes, genera, and the number of the cultivatable strains from *H. roretzi* stools**

| CLASS | GENUS | AEROBIC | | | | ANAEROBIC | TOTAL |
| --- | --- | --- | --- | --- | --- | --- | --- |
|  |  | SPRING | SUMMER | AUTUMN | WINTER |  |  |
| Actinomycetia | *Micrococcus* | 0 | 0 | 0 | 1 | 0 | 1 |
| Bacilli | *Acinetobacter* | 0 | 1 | 0 | 0 | 0 | 1 |
|  | *Alkalihalobacillus* | 1 | 0 | 1 | 5 | 0 | 7 |
|  | *Bacillus* | 11 | 13 | 20 | 27 | 32 | 103 |
|  | *Cytobacillus* | 0 | 1 | 0 | 1 | 0 | 2 |
|  | *Exiguobacterium* | 0 | 2 | 0 | 0 | 0 | 2 |
|  | *Lysinibacillus* | 0 | 8 | 0 | 4 | 0 | 12 |
|  | *Marinilactibacillus* | 0 | 0 | 0 | 0 | 3 | 3 |
|  | *Mesobacillus* | 1 | 0 | 0 | 0 | 0 | 1 |
|  | *Metabacillus* | 1 | 0 | 0 | 1 | 0 | 2 |
|  | *Neobacillus* | 0 | 0 | 0 | 4 | 0 | 4 |
|  | *Paenibacillus* | 0 | 0 | 0 | 4 | 0 | 4 |
|  | *Peribacillus* | 0 | 0 | 0 | 2 | 0 | 2 |
|  | *Solibacillus* | 1 | 0 | 0 | 0 | 0 | 1 |
|  | *Staphylococcus* | 0 | 0 | 0 | 0 | 19 | 19 |
| Clostridia | *Clostridium* | 0 | 0 | 0 | 0 | 3 | 3 |
|  | *Paraclostridium* | 0 | 0 | 0 | 0 | 8 | 8 |
|  | *Tepidibacter* | 0 | 0 | 0 | 0 | 13 | 13 |
|  | *Vallitalea* | 0 | 0 | 0 | 0 | 1 | 1 |
| Deltaproteobacteria | *Halodesulfovibrio* | 0 | 0 | 0 | 0 | 9 | 9 |
| Flavobacteriia | *Zunongwangia* | 0 | 0 | 0 | 0 | 1 | 1 |
| Gammaproteobacteria | *Aeromonas* | 0 | 0 | 1 | 0 | 0 | 1 |
|  | *Alcanivorax* | 0 | 0 | 0 | 0 | 1 | 1 |
|  | *Chromohalobacter* | 0 | 0 | 0 | 0 | 2 | 2 |
|  | *Citrobacter* | 0 | 1 | 0 | 0 | 4 | 5 |
|  | *Cobetia* | 0 | 0 | 1 | 0 | 0 | 1 |
|  | *Enterobacter* | 4 | 0 | 0 | 0 | 1 | 5 |
|  | *Klebsiella* | 0 | 0 | 0 | 0 | 1 | 1 |
|  | *Prolinoborus* | 0 | 0 | 2 | 0 | 0 | 2 |
|  | *Proteus* | 0 | 4 | 0 | 0 | 0 | 4 |
|  | *Providencia* | 1 | 0 | 0 | 0 | 0 | 1 |
|  | *Pseudoalteromonas* | 1 | 2 | 1 | 0 | 0 | 4 |
|  | *Pseudomonas* | 0 | 1 | 0 | 1 | 0 | 2 |
|  | *Serratia* | 16 | 0 | 0 | 7 | 0 | 23 |
|  | *Shewanella* | 0 | 0 | 2 | 0 | 0 | 2 |
|  | *Vibrio* | 2 | 4 | 2 | 2 | 0 | 10 |
| **total** | | **39** | **37** | **30** | **59** | **98** | **263** |
| **Shannon's diversity index** | | **2.65** | **2.591** | **2.792** | **3.0308** | **2.5611** | **3.96** |
| **Margalef's diversity index** | | **6.01** | **5.82** | **5.89** | **7.11** | **4.58** | **14.9** |
| **Menhiniek's diversity index** | | **3.52** | **3.45** | **3.47** | **3.78** | **2.12** | **5.12** |
| **Simpson's diversity index** | | **0.89** | **0.94** | **0.96** | **0.95** | **0.91** | **0.98** |
